# Supplementary figures and images for: Metabolome and transcriptome integration reveals insights into petals coloration mechanism of three species in Sect. Chrysantha chang
Source: PeerJ. 2024 Apr 19;12:e17275. doi: 10.7717/peerj.17275 (PMC11034495; doi:10.7717/peerj.17275)

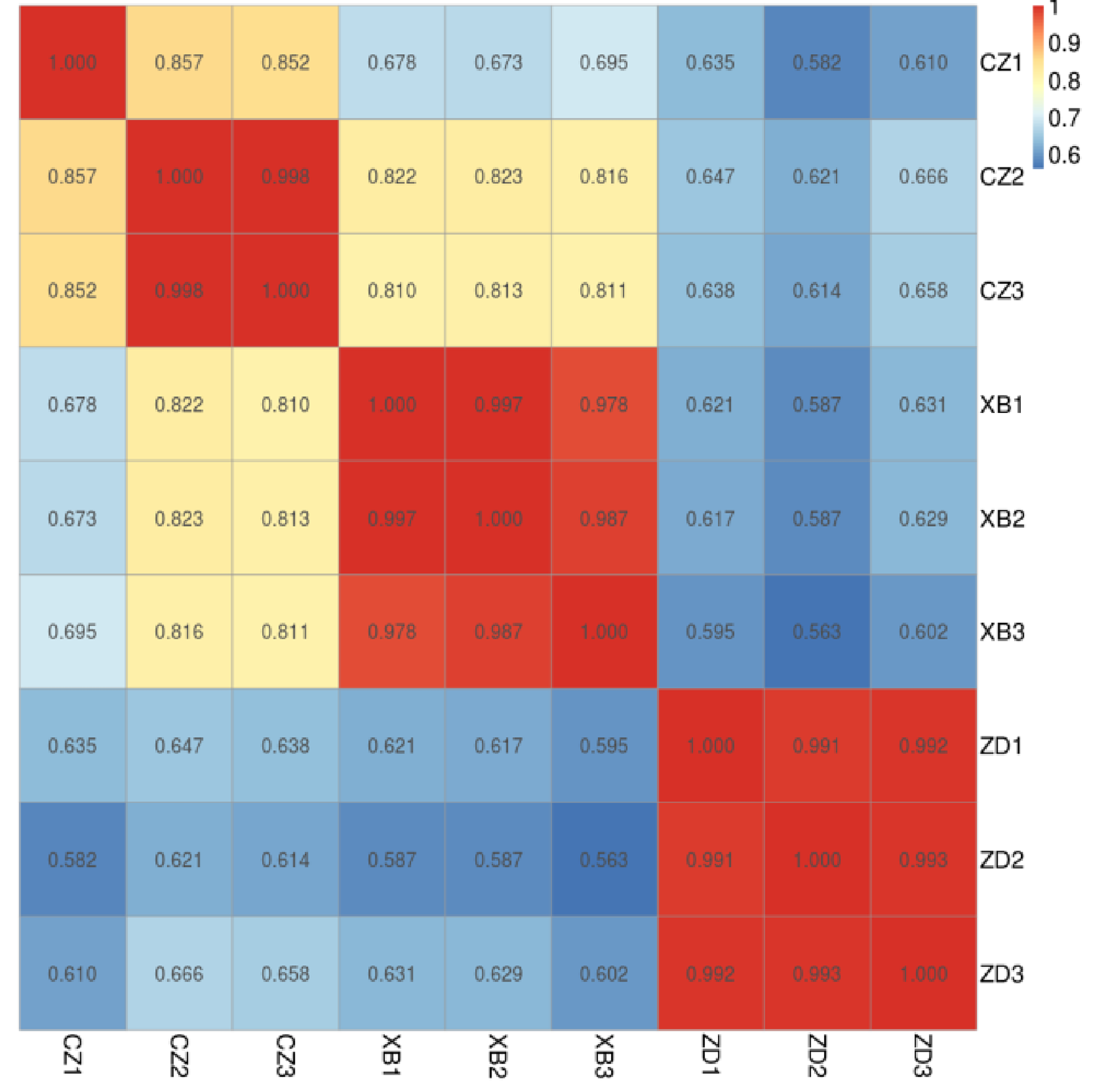

Supplement: Supplemental Information 1 [file peerj-12-17275-s001.png]
